# Magnetoencephalography in clinical practice

**DOI:** 10.1590/0004-282X-ANP-2021-0083

**Published:** 2022-04-22

**Authors:** Mohamed HEGAZY, Jay GAVVALA

**Affiliations:** 1 Baylor College of Medicine, Neurology, Houston, Texas, United States.

**Keywords:** Magnetoencephalography, Electroencephalography, Neurophysiology, Magnetic Resonance Imaging, Brain Mapping, Magnetoencefalografia, Eletroencefalografia, Neurofisiologia, Imagem por Ressonância Magnética, Mapeamento Encefálico

## Abstract

Magnetoencephalography (MEG) is a neurophysiological technique that measures the magnetic fields associated with neuronal activity in the brain. It is closely related but distinct from its counterpart electroencephalography (EEG). The first MEG was recorded more than 50 years ago and has technologically evolved over this time. It is now well established in clinical practice particularly in the field of epilepsy surgery and functional brain mapping. However, underutilization and misunderstanding of the clinical applications of MEG is a challenge to more widespread use of this technology. A fundamental understanding of the neurophysiology and physics of MEG is discussed in this article as well as practical issues related to implementation, analysis, and clinical applications. The future of MEG and some potential clinical applications are briefly reviewed.

## RESUMO

A magnetoencefalografia (MEG) é uma técnica neurofisiológica que mede os campos magnéticos associados à atividade neuronal no cérebro. A técnica é distinta da eletroencefalografia (EEG), porém está intimamente relacionada a ela. A primeira foi registrada há mais de 50 anos e evoluiu tecnologicamente ao longo do tempo. Está agora bem estabelecida na prática clínica, particularmente nos campos da cirurgia de epilepsia e mapeamento cerebral funcional. No entanto, a subutilização e a incompreensão das aplicações clínicas da MEG são um desafio para o uso mais amplo dessa tecnologia. Suas bases neurofisiológica e física são discutidas neste artigo, bem como questões práticas relacionadas à sua implementação, análise e aplicações clínicas. O futuro da MEG e algumas aplicações clínicas potenciais são brevemente revistos.

## INTRODUCTION

Electroencephalography (EEG is a neurophysiological technique that measures the electrical fields associated with neuronal activity in the brain^1^. Since the recording of the first human EEG by Hans Berger in 1924^2^, experience and understanding of the technology has evolved with EEG becoming widely utilized in clinical practice with widespread familiarity by neurologists. Magnetoencephalography (MEG) was recorded for the first time in 1968 by David Cohen^3^. Although the understanding, experience, and utility of MEG in clinical practice has evolved greatly since its inception, it remains less commonly used in clinical practice, and neurologists are less familiar with it as an important tool^4^. This article aims to familiarize neurologists and the clinical community at large with MEG and its applications in clinical practice.

## NEUROPHYSIOLOGICAL BASIS OF SIGNALS RECORDED FROM THE BRAIN

A common misconception about MEG in the clinical community is that MEG is considered as an imaging modality similar to MRI rather than a neurophysiological procedure. A likely reason is the confusion of MEG with magnetic source imaging (MSI). MSI is commonly utilized as a way to project MEG data on an MRI brain, but MEG itself doesn’t produce images of the brain and doesn’t involve emitting magnetic fields such as with MRI or any form of radiation. Source imaging is not unique to MEG, and it can also be applied to EEG (electrical source imaging (ESI)) and be used in combination with magnetic source imaging (electromagnetic source imaging, EMSI)^5^.

MEG is a neurophysiological technique that measures the magnetic fields associated with neuronal activity in the brain. It is closely related and complementary to EEG, with notable differences. While EEG uses electrodes to detect electrical activity from the brain, MEG uses special detectors (see below for more details) to measure the minute magnetic fields from the brain. Despite the fundamental differences in electrical and magnetic activity, both are nevertheless representations of the same underlying neuronal activity. Closer examination of this activity is fundamental to the understanding of both EEG and MEG^6,7^.

**Figure 1.**
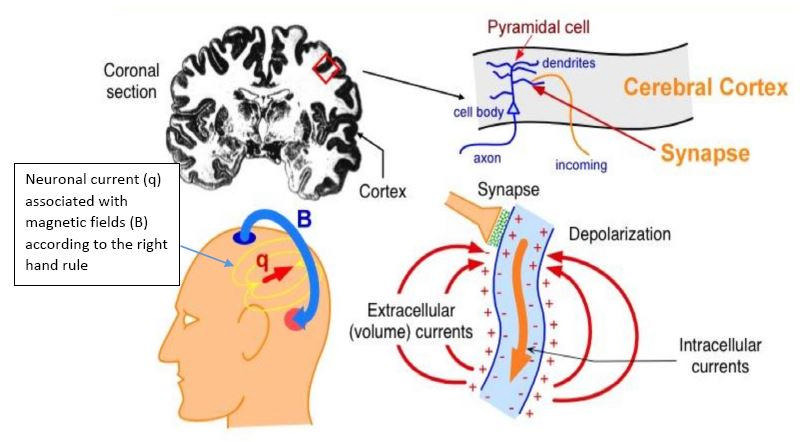
Basis of the signal detected by magnetoencephalography and electroencephalography: Section through the cerebral cortex showing a cortical pyramidal cell. Afferent signal occurring at the dendrite of the pyramidal cell results in changes in membrane potential with resultant primary intracellular current associated with a magnetic field that is detected by magnetoencephalography and secondary extracellular (volume) current that is detected by electroencephalography.

Source: http://ilabs.washington.edu/what-magnetoencephalography-meg. Accessed on: 14 Jun, 2021. MEG: magnetoencephalography; EEG: electroencephalography.

Neuronal activity in the brain is dependent on the electrochemical signaling between neurons that occurs at synapses, which causes a change in the post-synaptic membrane, resulting in a flow of ions across the membrane. Depending on the type of signal, these post-synaptic potentials can be either excitatory (EPSPs) or inhibitory (IPSP). These potentials result in an intra-neuronal primary current flow and secondary extracellular currents, which propagate through the various tissues (volume conduction) to reach the scalp where they can be detected by EEG electrodes and form the basis of the EEG recording. The primary intra-neuronal current is associated with a magnetic field, the direction of which is determined according to the right hand rule. This magnetic field is propagated outside the skull and can be detected by MEG sensors and forms the basis of MEG recording (Figure 1). It is important to recognize that the recording of these minute electric/magnetic fields is made feasible by the summation of these fields, which is achieved spatially due to the parallel orientation of pyramidal cells of the cortex and temporally by synchronous firing of multiple neurons^6,7^.

While the propagation of electrical activity depends on the presence of a medium (or volume conduction), magnetic fields do not require such a medium. Furthermore, each tissue type has a different conductivity for electrical activity, leading to distortion of the field as it passes through, whereas magnetic fields are unaltered by different tissues, which are essentially “transparent” to magnetic fields. This is a fundamental advantage of MEG over EEG when it comes to localization of the activity source within the brain. Furthermore, MEG demonstrates superior spatial resolution, with prior studies demonstrating that >10 cm^2^ of cortex must be activated to generate a detectable scalp EEG signal, while for MEG about 6 cm^2^ of cortex has to be activated^6,7^.

**Figure 2.**
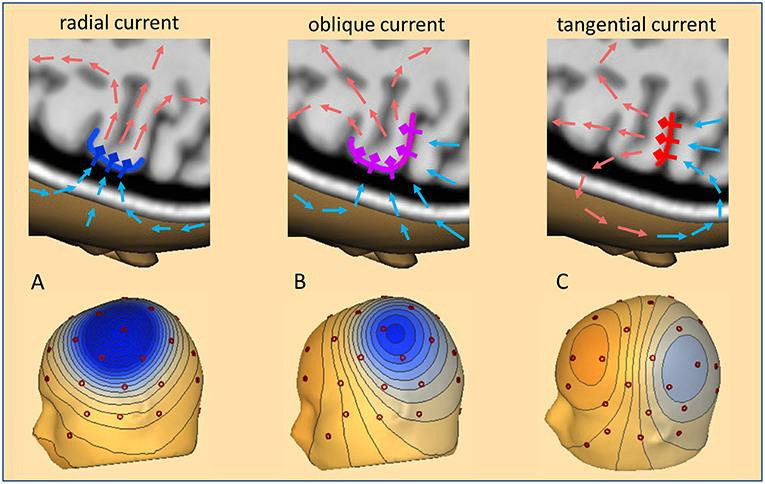
Source orientation A shows a radial source at the crown of a gyrus perpendicular to the skull with maximal negativity (blue on the topography map) detected by electrodes directly over the source. electroencephalography can preferentially detect this type of source while magnetoencephalography is insensitive to this activity. Example B shows an oblique source that is intermediate in orientation between a radial and tangential source. Example C shows a tangential source in the depth of sulcus parallel to the skull with dipolar field (blue and red on the topography map) and the location of the source in between the 2 maxima. Magnetoencephalography can preferentially detect this type of source.

Source: Sherg et al.^21^. MEG: magnetoencephalography; EEG: electroencephalography.

One important concept to consider is source orientation (Figure 2). This refers to the orientation of the cortical source of the electro-magnetic signal (orientation of the pyramidal cells and the direction of the resulting current directed from the dendrites towards the soma) in relation to the skull. A source is said to be tangential to the skull when it is oriented parallel to the skull and includes cortical sources that lie within the sulci of the brain. Such tangential sources produce magnetic fields according to the right-hand rule that are perpendicular to the electric current and therefore propagate outside the skull and are best suited for detection by MEG. In contrast, radial sources are oriented perpendicular to the skull (cortex of the crown of gyri) and produce electric currents with magnetic fields that propagate in such a way that they are less likely to be detected by MEG, but because the electric current is directed outward, it is ideally detected by EEG^6,7^.

**Figure 3.**
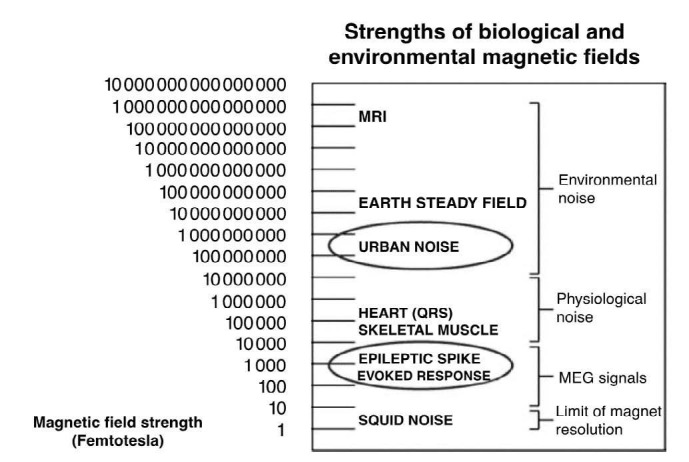
Comparative magnetic strength of different signals.

Source: Papanicolaou^7^. SQUID: Superconducting Quantum Interference Device; MEG: magnetoencephalography; MRI: magnetic resonance image.

**Figure 4.**
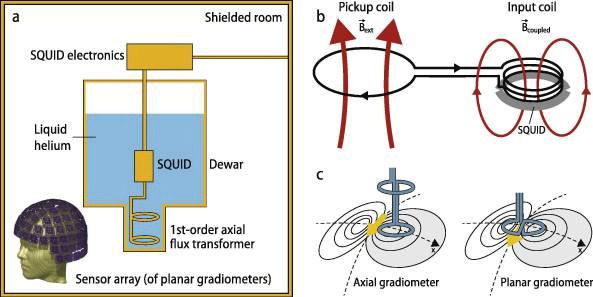
Typical magnetoencephalography system consisting of a flux transformer that detects the magnetic field, coupled to a Superconducting Quantum Interference Device cooled with liquid helium inside a Dewar and housed within a magnetically shielded room.

Source: Hari et al.^20^. SQUID: Superconducting Quantum Interference Device.

There are several other important technical differences between MEG and EEG. EEG utilizes electrodes that detect electric potentials and have to be directly applied to the skull and require a reference. MEG utilizes special sensors that detect minute magnetic fields (see Figure 3 for comparative magnetic strength of different signals), which are confined within a helmet and therefore are not in direct contact with the skull and require no reference. MEG sensors rely on the physical phenomenon of superconductivity to detect the extremely minute magnetic fields produced by the brain with extremely low temperatures near zero K. This is achieved using SQUIDs (superconducting quantum interference devices), which are cooled using liquid helium in a Dewar container coupled to flux transformers that sense the magnetic field. Flux transformers consist of either a single superconducting coil (magnetometers) or 2 oppositely wound coils (gradiometers). Gradiometers are designed such that the 2 coils are either placed one above the other (axial) or next to each other (planar) and have the advantage of being less affected by external noise than magnetometers. MEG sensors are arranged in the shape of a helmet that covers the skull with several hundred sensors, a significantly more than a typical EEG system. MEG’s sensors are housed in a magnetically shielded room (MSR), which functions as a shield against external magnetic noise. As a result, MEG machines require a dedicated room and are expensive to purchase and maintain, limiting widespread adoption of this technology^6,7^ (Figure 4).

## MAGNETOENCEPHALOGRAPHY WORKFLOW

While the process of acquiring an EEG can be relatively simple, the process of preparation, recording, and analysis of MEG can be more complex and labor-intensive. In preparation for MEG implementation, sources of magnetic noise need to be eliminated as much as possible. Some of these sources are removable, such as accessories, cell phones etc., but some are not, such as implanted devices like neuromodulatory devices (Vagus nerve stimulators or implanted dental hardware). After careful survey and removal of sources of magnetic noise in a subject, residual magnetic artifacts can be removed from a subject using demagnetizing equipment in a process called degaussing and after recording using special software. Obtaining an EEG during a MEG recording to optimize analysis and interpretation is a standard practice^8^. A crucial step is the application of head position indicator coils and head-shape digitization device, which are essential for co-registration of MEG data during the patient’s brain MRI process by magnetic source imaging. A typical MEG recording is about 60-120 minutes long. Additional time may be necessary if functional mapping is requested, such as somatosensory or language mapping^9^. In some centers, particularly in pediatric cases, sedation is used to ensure that the patient is lying still^8^. After data acquisition, data are further processed for artifact removal and motion correction. The final step is data analysis by a magnetoencephalographer and preparation of a report^10^.^11^.

## MAGNETOENCEPHALOGRAPHY DATA ANALYSIS

**Figure 5.**
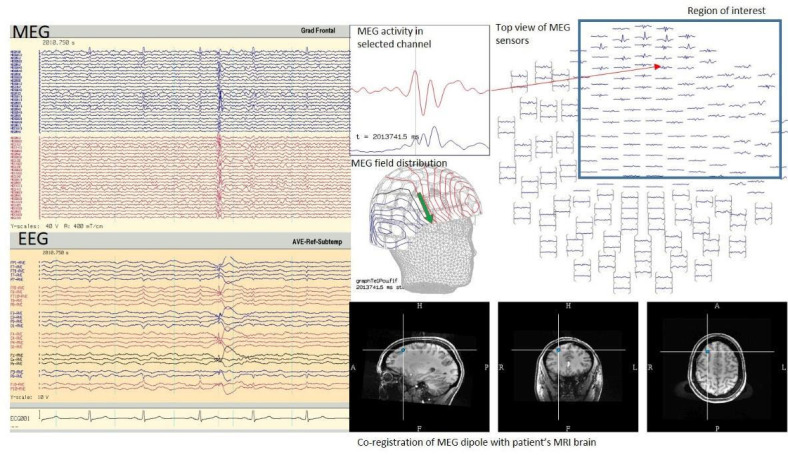
Tracings from magnetoencephalography at the top and from electroencephalography at the bottom and typical presentation of magnetoencephalography activity with magnetic field distribution and co-registration with the patient’s brain magnetic resonance image.

MEG: magnetoencephalography; EEG: electroencephalography; MRI: magnetic resonance image.

Raw MEG data is displayed similarly to EEG in the form of channels, each representing a MEG sensor, and waveforms plotted against time. As mentioned previously, EEG is recorded simultaneously, and both datasets are also analyzed simultaneously. Similar to EEG analysis, visual analysis of waveforms is a cornerstone of MEG data analysis to identify normal and abnormal waveforms such as epileptiform discharges. Once an abnormal waveform is identified, the process of source imaging is used to display the estimated location of the source of the activity on the patient’s brain MRI (Figure 5). Similar to EEG, it is important to note the presence of normal variations in MEG and to avoid the misinterpretation of these variations as abnormal^12^.

The process of source imaging is a computational model that uses the measured magnetic field of the head to estimate the location of the source of that activity in the brain (inverse problem). For this, a model of the source of the activity and a model of the head within which the source is contained are needed. In clinical MEG practice, the head is typically modelled as a sphere. The source model most commonly used in clinical practice is the equivalent current dipole (ECD). This model assumes the source of activity to be a point represented by a dipole with 2 ends, a positive end (current source) and a negative end (current sink). The obvious limitation of this model is that the actual cortical sources of activity are more complex and are not just a point in space. However, the simple computational processing and relative ease of interpretation have made this model the most commonly used method of analysis. Other source models exist, such as distributed source and beamformer models, but they are beyond the scope of this article^12^.

## MAGNETOENCEPHALOGRAPHY IN CLINICAL PRACTICE

There are currently 2 well-established uses of MEG in clinical practice. The first is for the localization of epileptic activity in patients with drug-resistant epilepsy. The second is for the localization of eloquent cortex as pre-surgical planning for patients undergoing a resective neurosurgery.

Assessment of eligibility for epilepsy surgery in drug-resistant epilepsy involves a non-invasive evaluation that usually includes video EEG, brain MRI, brain PET, SPECT, and neuropsychological testing. In some cases, a surgical procedure can be offered at this stage. However, it is not uncommon for non-invasive data to be inconsistent and invasive evaluation with intracranial EEG is needed (either stereo-EEG or subdural grid). MEG can influence the process of epilepsy surgery in several ways. In about one-third of cases, MEG provides important, non-redundant information that can affect the surgical decision-making process^11^. This information can guide the implantation plan for an intracranial EEG and positively impact surgical outcome. This is especially true when planning a stereo-EEG, where there is limited spatial sampling based on the properties of the electrodes^13^. In some cases, the information provided by MEG allows patients to skip intra-cranial EEG and proceed directly to surgery, whereas in others cases, MEG data have let to patients previously thought to be ineligible for surgery becoming surgical candidates^14,15^.

The value of MEG in epilepsy surgery is attributed to the nature of the technology, which provides particular advantages in detecting sources of epileptic activity that are not optimally recorded by EEG. As a general rule, MEG will preferentially detect epileptic activity arising from sulci in the brain. Common examples where MEG has been shown to be effective include the peri-sylvian, operculo-insular, mesial-frontal, and interhemispheric regions. Another clinical scenario in which MEG is beneficial is MRI-negative epilepsy with suspected origin in the mesial temporal lobe, where MEG can detect spikes in the mesial temporal lobe. Other clinical scenarios are epilepsies associated with multiple brain lesions, such as tuberous sclerosis (TS), in which MEG can help identify the “dominant” tuber, and large brain lesions such as large or hemispheric cortical malformations, in which MEG can identify the most active part of the lesion. In patients with epilepsy with prior craniotomy and seizure recurrence after surgery who are being evaluated for further surgical intervention, the skull defect and anatomical changes can lead to distortion of EEG findings and interpretation, while MEG findings are unaltered by these changes^14,15^. The indications of MEG in epilepsy surgery are summarized in Table 1.

**Table 1.** Indications of magnetoencephalography in epilepsy surgery.

Clinical scenarios in which MEG is indicated
MRI negative (non lesional) epilepsy of suspected medial temporal origin
Perisylvian and insular epilepsy
Interhemispheric epilepsy (e.g., mesial frontal or mesial occipital)
Epilepsies associated with multiple brain lesions, such as tuberous sclerosis (TS), or with large brain lesions, such as large or hemispheric cortical malformations
Evaluation of seizure recurrence after craniotomy for epilepsy surgery

MEG: magnetoencephalography; MRI: magnetic resonance image.

## MAGNETOENCEPHALOGRAPHY IN FUNCTIONAL BRAIN MAPPING

In addition to recording spontaneous brain activity, including abnormal epileptiform activity, MEG is capable of recording evoked magnetic fields to aid in localization of the eloquent cortex. This offers a non-invasive way of establishing the relationship of various brain lesions to the eloquent cortex prior to neurosurgical intervention. While fMRI is commonly used to identify sensory-motor cortices, MEG has higher temporal resolution. Several methods have been used, but electrical stimulation of peripheral nerves or tactile stimulation commonly generate somatosensory evoked fields^16^. Auditory evoked fields are reliably generated in primary auditory cortex within the Sylvian fissure with 100 ms deflections^17^. Visual evoked fields can identify the eloquent occipital cortex with reproducible waveforms at 100 ms^18^. Language lateralization has been performed via MEG with several methodologies, each with comparable efficacy to the Wada or fMRI^19^. Using MSI, recorded responses are analyzed and displayed on the patient’s brain MRI.

## FUTURE OF MAGNETOENCEPHALOGRAPHY AND POTENTIAL APPLICATIONS

On the technical side, there are advances that would make MEG more affordable and accessible. Optically pumped magnetometers (OPM) can be positioned directly to the patient’s head and do not require cooling, reducing some of the maintenance costs. Furthermore, these sensors allow for better portability. Improvements in MEG data processing, including the possibility of automated workflows, are under development and promise to reduce the labor-intensive nature of MEG analysis.

Several clinical research studies are currently underway using MEG as a biomarker, including identifying neurophysiological biomarkers for Alzheimer’s disease and exploring efficacy of treatment for obsessive-compulsive disorder. Table 2 summarizes some of the diseases in which MEG may have a clinical role perhaps not so distant in the future^20^.

**Table 2.** Possible future clinical applications of magnetoencephalography.

Potential role of MEG in some neuropsychiatric diseases in the future
Stroke
Alzheimer’s disease
Traumatic brain injury
Chronic pain
Parkinson’s disease
Schizophrenia
Autism spectrum disorders
ADHD

MEG: magnetoencephalography; ADHD: attention deficit hyperactivity disorder.

In conclusion, MEG has become established in clinical practice particularly in epilepsy surgery and functional brain mapping. There is accumulating evidence of its usefulness in clinical practice, but it remains underutilized and poorly understood even within the clinical community. Higher awareness and formal education about MEG are needed, and, as technology advances and costs decrease, it is expected that MEG will become more widely used and have more applications.
